# A Sparsity-Constrained Preconditioned Kaczmarz Reconstruction Method for Fluorescence Molecular Tomography

**DOI:** 10.1155/2016/4504161

**Published:** 2016-11-24

**Authors:** Duofan Chen, Jimin Liang, Yao Li, Guanghui Qiu

**Affiliations:** Life Science and Technology, Xidian University, Xi'an, Shaanxi 710071, China

## Abstract

Fluorescence molecular tomography (FMT) is an imaging technique that can localize and quantify fluorescent markers to resolve biological processes at molecular and cellular levels. Owing to a limited number of measurements and a large number of unknowns as well as the diffusive transport of photons in biological tissues, the inverse problem in FMT is usually highly ill-posed. In this work, a sparsity-constrained preconditioned Kaczmarz (SCP-Kaczmarz) method is proposed to reconstruct the fluorescent target for FMT. The SCP-Kaczmarz method uses the preconditioning strategy to minimize the correlation between the rows of the forward matrix and constrains the Kaczmarz iteration results to be sparse. Numerical simulation and phantom and in vivo experiments were performed to test the efficiency of the proposed method. The results demonstrate that both the convergence and accuracy of the proposed method are improved compared with the classical memory-efficient low-cost Kaczmarz method.

## 1. Introduction

Fluorescence molecular tomography (FMT) is an imaging modality that can localize and quantify fluorescent markers to resolve biological processes at molecular and cellular levels. Being minimally invasive, relatively inexpensive, and portable, FMT has been successfully applied in small animal research and preclinical diagnostics such as cancer diagnosis, drug discovery, and gene expression visualization [1–4].

Due to a large number of unknowns and a limited number of measurements as well as the diffusive transport of photons in biological tissues, FMT reconstruction is an ill-posed inverse problem [5–7]. To improve the FMT imaging quality, both the noncontact FMT technique [8, 9] and the strategy of multiple excitations can be used to obtain more measurements. Structural a priori information provided by CT or MRI can also be incorporated into FMT imaging [3, 10–12]. Moreover, reconstruction algorithms can resort to regularization strategies and find meaningful and numerically stable solutions. In [13, 14], the Tikhonov regularization, namely, *l*_2_ norm regularization, is employed for solving the inverse problem. In [15–17], the sparsity regularization is utilized in the form of *l*_1_ norm penalty function for FMT reconstruction. Joint *l*_1_ and TV regularization for FMT reconstruction is presented in [18]. In [19], a hybrid regularization method incorporating *l*_1_ and *l*_2_ norm penalty is proposed to recover the 3D fluorophore distribution. In these techniques, optimal selection of the regularization parameter is needed to avoid over- or underregularization. Being a memory-efficient low-cost numerical solver that avoids bulky matrix computations in large-scale problems, Kaczmarz algorithm, also known as algebraic reconstruction technique (ART), iteratively updates the solution using only one equation at a time and has been applied in optical tomographic reconstruction [20–22]. During reconstruction, the Kaczmarz method may use the measurements in the order that they are collected, which is known as the sequential access order. To speed up the convergence rate of the Kaczmarz method and give better results in the first iteration relative to the sequential access scheme, different access orders have been proposed [23–25]. The idea of these different access orders is to minimize the correlation between measurements that are successively accessed by the iterative projection inversion method. In [20], the influence of the data access order is investigated when Kaczmarz method is used to perform diffuse optical tomography. The study shows that the convergence speed can be significantly improved by selecting proper projection access order.

In FMT, the forward matrix maps the fluorescent targets to the surface measurements. Generally, the rows of the forward matrix are correlated because of the correlations among source-detector maps from the same projection and the interrelations of different projections [26, 27]. In this work, we present a strategy which computes a preconditioning matrix to minimize the coherence of the preconditioned forward matrix. Then the Kaczmarz method which uses the sequential access order is adopted to solve the preconditioned FMT reconstruction problem. After preconditioning, the projections are close to perpendicularity and the convergence rate of the Kaczmarz method can be speeded up. As most optical fluorophores are designed to accumulate in relatively small, specific regions in tissues, such as tumors, and hence the fluorophore distributes sparsely in the imaging domain, we propose sparsity-constrained reconstruction method to perform FMT and the method is named as sparsity-constrained preconditioned Kaczmarz (SCP-Kaczmarz) method. Different from the existed *l*_1_ norm regularization methods, this proposed SCP-Kaczmarz method adopts a thresholding step to the Kaczmarz results to satisfy a user-defined sparsity value.

The remaining of this paper is organized as follows. We first describe the mathematical forward model for FMT imaging, then the SCP-Kaczmarz method is presented for FMT reconstruction, then the numerical simulation and physical phantom and in vivo experiments are performed to evaluate the proposed method, and finally the discussion and conclusion are given.

## 2. Methods

### 2.1. Forward Model for FMT Imaging

When a CW point laser is used as excitation light, the diffusion of excitation and emission lights through biological tissues can be described by two coupled diffusion equations with the Robin-type boundary condition, and the coupled diffusion equations can be presented as follows [28]:

(1) ∇·Dxr∇Φxr−μaxrΦxr=−Θδr−rS,∇·Dmr∇Φmr−μamrΦmr=−Φxrημafr,

where *r* ∈ *Ω*, *Ω* being the domain under consideration. The subscripts *x* and *m* denote excitation light and emission light, respectively. *D*_*x*,*m*_ = 1/3(*μ*_*ax*,*am*_ + (1 − *g*)*μ*_*sx*,*sm*_) is the diffusion coefficient with *μ*_*sx*,*sm*_ as the scattering coefficient, *g* is the anisotropy parameter, and *μ*_*ax*,*am*_ is the absorption coefficient. Φ_*x*,*m*_ denotes the photon density. The fluorescent yield *ημ*_*af*_ is the unknown parameter to be reconstructed, which is denoted as *x* hereafter. By using the finite element method (FEM), the linear relationship between the boundary measurements *ϕ*_*m*_ and the desired unknown fluorophore distribution *x* can be obtained from (1) and is described by

(2) $$\begin{array}{c} \phi_{m}=Ax, \end{array}$$

where **A** is the forward matrix, the sizes of *ϕ*_*m*_, **A**, and *x* are *M* × 1, *M* × *N*, and *N* × 1, respectively. *M* is the number of surface measurements and *N* is the number of unknowns needed to be determined inside the imaging domain. Usually *M* < *N*, and this means that the number of measurements is smaller than that of the unknowns.

### 2.2. Sparsity-Constrained Preconditioned Kaczmarz Method

It is known to us that the convergence of the Kaczmarz method is affected by the data access order. If the measurements are prearranged in a scheme that the projections are close to perpendicularity, the convergence of the Kaczmarz method will be speeded up. In this paper, rather than changing the sequential data access order, we design a preconditioner to minimize the correlation between the rows of the forward matrix of FMT and hence to make the Kaczmarz method converge quickly. Denote the preconditioning matrix as **W** and the preconditioned forward matrix as **B**, then we get **B** = **W** **A**, and **W** can be obtained by solving the following optimization problem:

(3) min BBT−IMF=minW WAATWT−IMF,

where **I**_*M*_ is the *M* × *M* identity matrix and ‖·‖_F_ is the Frobenius norm.

Considering the singular value decomposition of **A** which is described by **A** = **U** **S** **V** ^T^, where **U** is *M* × *M* unitary matrix, **S** is *M* × *N* diagonal nonnegative matrix and **V** is an *N* × *N* unitary matrix. Letting **W** = (**S** **S** ^T^)^−1/2^ **U** ^T^, we can get

(4) BBTWAATWT=SST−1/2UTUSVTVSTUTUSST−1/2=IM.

Equation (4) means that the rows of the preconditioned forward matrix are orthogonal to one another. If the preconditioner **W** is badly conditioned, we can use the diagonal loading strategy to mitigate the ill condition and **W** is calculated by **W** = (**S** **S** ^T^ + *λ* **I**)^−1/2^, where *λ* ≪ 1 is a constant [27].

**Figure 1:**
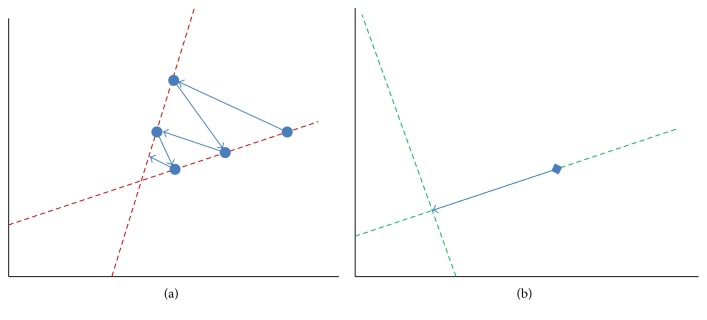
Geometric interpretations of the Kaczmarz algorithm and the preconditioned Kaczmarz algorithm applied to a 2D problem. (a) The red dashed lines represent the two equations in the 2D solution space; the blue dots and arrows show the convergence of the Kaczmarz algorithm to the solution. (b) The green dashed lines represent the two preconditioned equations in the 2D solution space; the blue diamond and arrow show the convergence of the preconditioned Kaczmarz algorithm to the solution.

Figure 1 provides a geometric insight into the iterative progress of the Kaczmarz and the preconditioned Kaczmarz algorithms.  Figure 1(a) presents a geometrical interpretation of Kaczmarz applied to a 2D problem. Here, each line represents a hyperplane in the solution space corresponding to one of the equations, and the solution is the intersection of the dashed lines. The progress of Kaczmarz is represented by dark blue dots and arrow lines. As depicted in Figure 1(a), the points with dots iteratively progress toward the solution (intersection of the two dashed lines) by orthogonal successive projections onto the two lines [22]. In Figure 1(b), the blue diamond and arrow demonstrate the convergence of preconditioned Kaczmarz algorithm toward the solution. Because the forward matrix has been preconditioned, the two green dashed lines which demonstrate the hyperplanes corresponding to the two equations are perpendicular. In theory, only one iteration is needed for the algorithm to converge to the solution. However, because of the ill condition of the forward matrix in FMT imaging and the presence of noise, the two lines are not completely perpendicular.

Multiplying (2) by **W** on both sides, we can obtain the distribution of the fluorescent targets by solving

(5) $$\begin{array}{c} Bx=W\phi_{m}. \end{array}$$

We use the classical Kaczmarz technique to solve (5) and the unknown *x* is updated by

(6) $$\begin{array}{c} x^{\left(k\right)}=x^{\left(k-1\right)}+B_{i}^{T}\frac{W\phi_{m}-B_{i}x^{\left(k-1\right)}}{B_{i}B_{i}^{T}},\;i=1,2,\ldots ,M, \end{array}$$

where **B**_*i*_ is the *i*th row of **B**.

Considering that the fluorescent target is sparsely distributed, we add a thresholding step to (6) to make the sparsity of the Kaczmarz result remain as close as possible to a preset value. The thresholding procedure is depicted by

(7) xnk=xnkif  xnk≥β maxxk0otherwise,n=1,2,…,N,

where *x*_*n*_^(*k*)^ is the *n*th element of *x* ^(*k*)^ and *β* is between 0 and 1 and can be obtained by solving the minimization problem [29]

(8) $$\begin{array}{c} \beta =\arg\;\min\left|\;sparsity\left(\;x^{\left(k\right)}\left(\;\beta \;\right)\;\right)-\psi \;\right|. \end{array}$$

**Figure 2:**
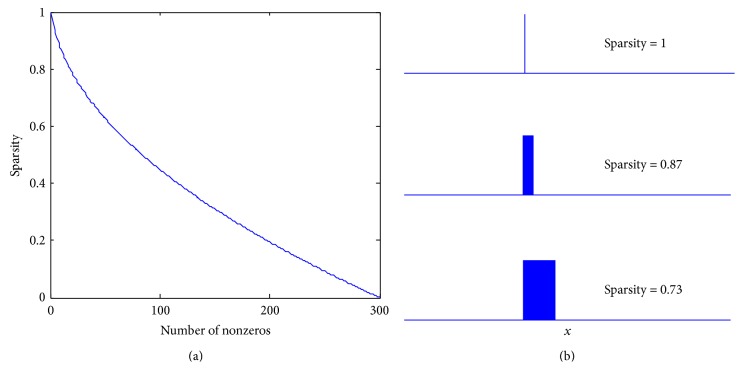
(a) The variation of sparsity value with the number of nonzero elements in *x*. (b) *x* with sparsity value of 1, 0.87, and 0.73.

The thresholding step also guarantees the nonnegativity of the solution. The minimization problem of (8) can be solved by using linear searching strategy. And the sparsity of *x* in (8) is defined as [30]

(9) $$\begin{array}{c} sparsity\left(\;x\;\right)=\frac{\sqrt{N}-\left({\left\|x\right\|}_{1}/{\left\|x\right\|}_{2}\right)}{\sqrt{N}-1}, \end{array}$$

where *N* is the size of vector *x* and ‖*x*‖_1_ and ‖*x*‖_2_ denote *l*_1_ and *l*_2_ norm of *x*, respectively. The curve in Figure 2(a) depicts the variation of sparsity value with the number of nonzero elements in *x* (assume that *x* has 300 elements and the nonzeros in *x* are constant, e.g., 1), from which we can see that the sparsity value ranges from 0 for nonsparse results to 1 for extremely sparse results.  Figure 2(b) shows *x* with sparsity value of 1, 0.87, and 0.73, respectively.

So far, the implementation of the proposed SCP-Kaczmarz method can be summarized as follows:

(1) Initialize *x* ^(*k*)^, where *k* = 0, and preset the wanted sparsity value *ψ*.
(2) Perform singular value decomposition to** A** and compute the preconditioning matrix **W**.
(3) Compute the preconditioned measurements *y* = **W** *ϕ*_*m*_ and the preconditioned forward matrix **B** = **W** **A**.
(4) Update *x* ^(*k*)^ from *x* ^(*k*−1)^ by solving *y* = **B** *x* using the classical Kaczmarz method.
(5) Keep the large elements of *x* ^(*k*)^ and set the other elements to zero to make the sparsity of the result equal to the wanted sparsity value *ψ*.
(6) Increase the iteration index *k* by 1 and set *x* ^(*k*)^ to be the initial value; repeat steps (4) to (5) until the stop criterion is achieved (e.g., when *k* = *K*_iter_ or ‖*x* ^(*k*)^ − *x* ^(*k*−1)^‖_2_ < *ε*).

A parameter, named as the wanted sparsity value *ψ*, should be predetermined for the proposed SCP-Kaczmarz method. As we do not know the true distribution of the fluorescent target in practice, the ratio of the volume of the fluorescent target to that of the imaging domain can be first estimated, and then the corresponding sparsity is calculated as the wanted *ψ* according to (9) under the assumption that the fluorescent target is uniformly distributed.

### 2.3. Experiments and Results

To demonstrate the performance of the proposed method, numerical simulation and phantom and in vivo experiments were conducted. In these experiments, cases with single fluorescent target and multiple fluorescent targets were considered. All the reconstructions were implemented on a personal computer with an 8 GB memory and an Intel-Core i7 CPU. The relative deviation, Dice coefficient, and sparsity value were calculated to evaluate the SCP-Kaczmarz algorithm. The relative deviation is defined by *δ* = ‖*x*_*r*_ − *x*_*t*_‖_2_/‖*x*_*t*_‖_2_, where *x*_*r*_ is the reconstructed target and *x*_*t*_ is the actual target. The Dice coefficient is defined by *D* = 2‖*x*_*r*_ · *x*_*t*_‖_2_/‖*x*_*r*_‖_2_^2^‖*x*_*t*_‖_2_^2^, where · is Hadamard product and the sparsity is defined by (9). In addition, the computational time and memory consumption of both the methods were also recorded.

### 2.4. Numerical Simulation Experiments

In the numerical simulation, a 3D digital mouse atlas of CT and cryosection data was utilized to provide anatomical information [31]. Cases with single, two, and three fluorescent targets inside the mouse atlas were studied, respectively.

#### 2.4.1. Reconstruction of Single Fluorescent Target

In this section, one fluorescent target inside the digital mouse atlas was reconstructed and two imaging models were investigated. The first model is reconstruction of small fluorescent target while the second one is reconstruction of big fluorescent target. The small target model is usually used to mimic small tissue with fluorescent probe, such as the early tumor; the big target model can be used to recover the biodistribution of fluorescent probe in organs, which is important in drug pharmacokinetics study [32].

**Figure 3:**
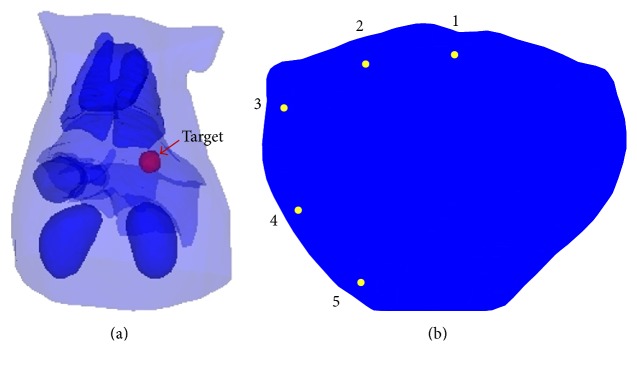
(a) The digital atlas torso used in the numerical simulation. (b) The plane of the 5-point excitation sources at *z* = 16.5 mm.

The small target model is shown in Figure 3(a), where a spherical fluorescent target (marked in red color) with radius of 1.5 mm was placed at “12.9 mm, 9.9 mm, and 16.5 mm” in the liver. The optical parameters of the mouse organs including muscle, heart, lungs, liver, kidneys, and stomach were listed in Table 1 [19]. As illustrated in Figure 3(b), the fluorescent target was excited sequentially by 5-isotropic point sources located one mean free path of photon transport beneath the mouse surface on *z* = 16.5 mm plane. For inverse reconstruction, the atlas torso was discretized into 38735 tetrahedrons and 7511 nodes. The sparsity value and fluorophore distribution were set to be 0.9 and 0, respectively, when *k* = 0.

**Figure 4:**
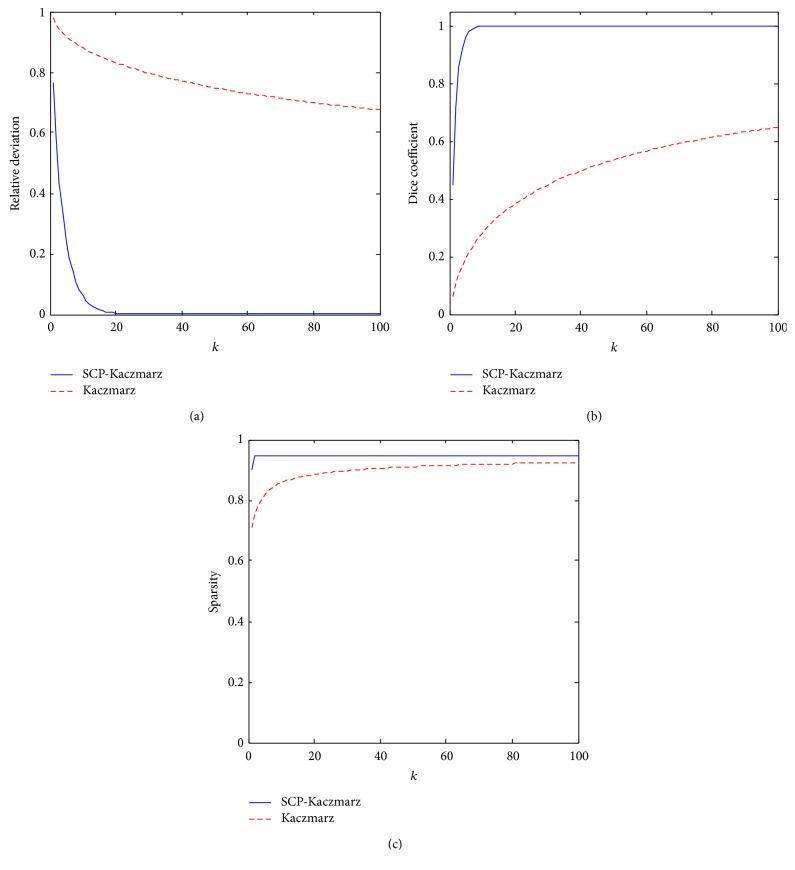
Results obtained by the SCP-Kaczmarz method (blue solid curves) and the Kaczmarz (red dashed curves) method. (a) The relative deviation. (b) The Dice coefficient. (c) The sparsity of the reconstructed target.

Figure 4 shows the relative deviation, the Dice coefficient, and the sparsity value obtained during the iteration process of the SCP-Kaczmarz and Kaczmarz algorithm. From Figure 4 we can see that, after about 20 iterations, the proposed method converges to the true solution with sparsity value of 0.9476.

**Figure 5:**
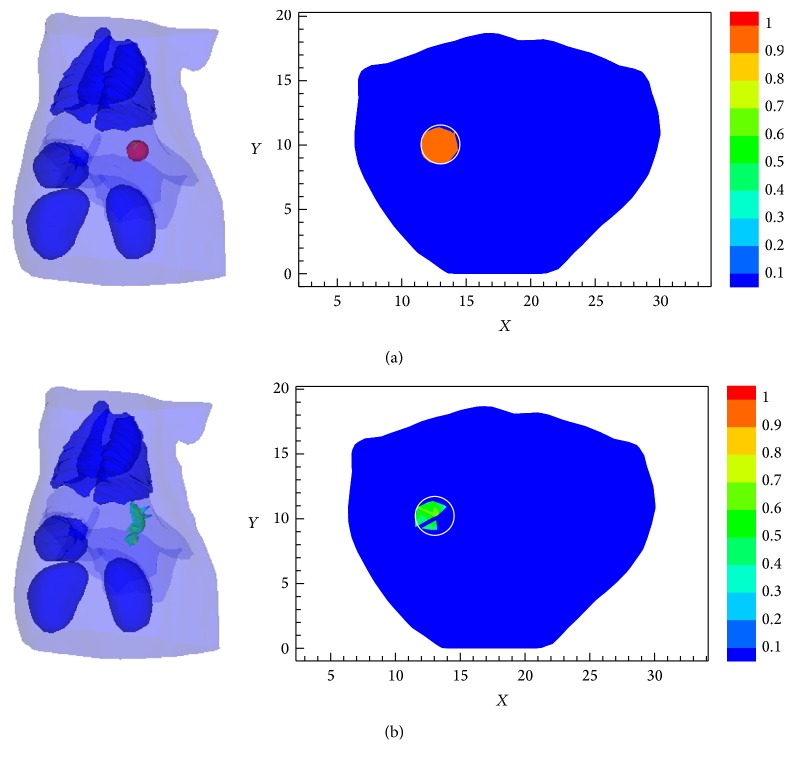
The reconstructed results of single small target. (a) 3D fluorescent targets and 2D slices at *z* = 16.5 mm obtained by the SCP-Kaczmarz method. (b) 3D fluorescent targets and 2D slices at *z* = 16.5 mm obtained by the Kaczmarz method.

Figure 5 shows the 3D targets and 2D slices at *z* = 16.5 mm recovered by the SCP-Kaczmarz and Kaczmarz method after 100 iterations, respectively. The actual target is indicated by the black circle and the reconstructed results are normalized by the true intensity. It can be seen that both methods can locate the target accurately, while the first one is more appropriate for quantitative analysis and profile reconstruction. It is known to us that the singular value decomposition of the forward matrix, needed for the proposed method to calculate the preconditioner, is computationally expensive. Fortunately, the SVD only needs to be performed for one time and can be done before the iteration starts. So, in this work, we just measure the elapsed time for the proposed algorithm to iteratively solve the preconditioned FMT inverse problem by using the MATLAB functions tic and toc and the SVD time cost is not included. The time cost is 79 seconds and 100 seconds, respectively, for the SCP-Kaczmarz method and the Kaczmarz method to run 100 iterations. The corresponding memory consumption, which is calculated by using the MATLAB instruction profile on memory, is 565040 KB and 565016 KB. As the memory is mainly used to store the preconditioned or the original forward matrix, the two algorithms have similar memory cost.

**Figure 6:**
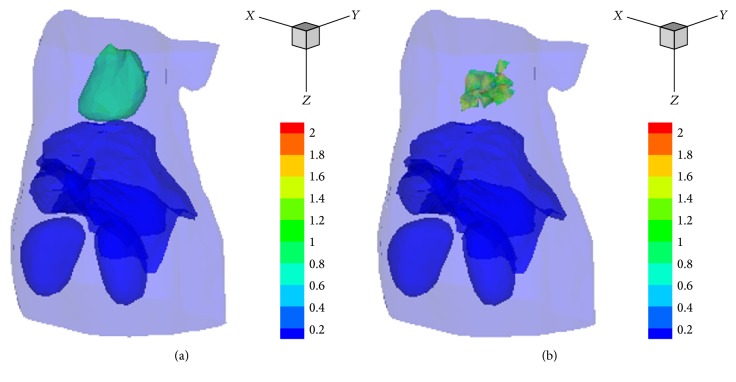
The reconstructed 3D results for big target model. (a) Results obtained by the SCP-Kaczmarz method. (b) Results obtained by the Kaczmarz method.

**Figure 7:**
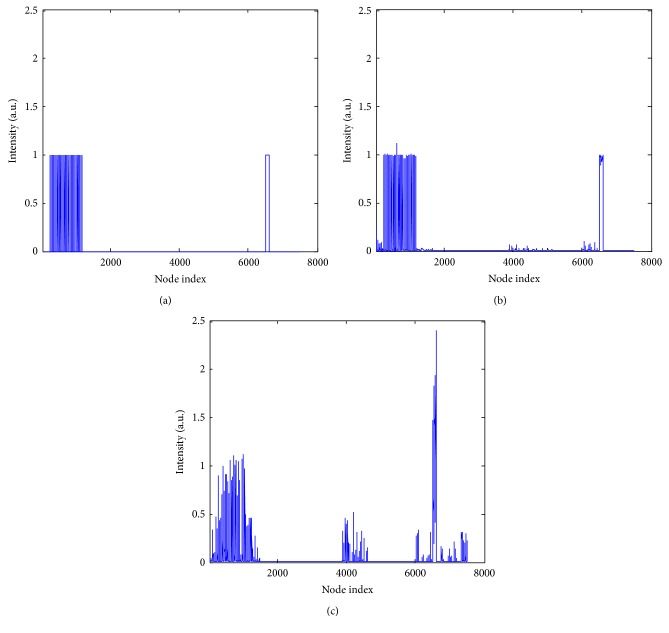
Intensity distribution inside the discretized atlas torso. (a) The true intensity distribution. (b) The recovered intensity by SCP-Kaczmarz method. (c) The recovered intensity by Kaczmarz method.

**Figure 8:**
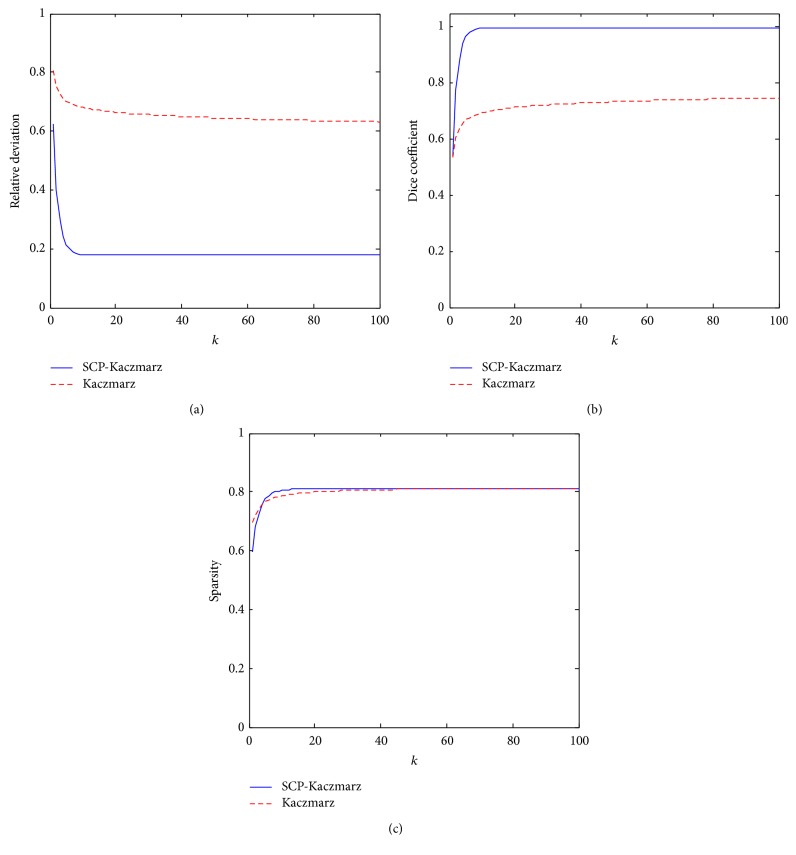
The recovered results by the two methods with 100 iterations. (a) The relative deviation. (b) The Dice coefficient. (c) The sparsity.

The big target model was used to recover the biodistribution of fluorescent probe in heart. The target was excited sequentially by 5-isotropic point sources located at one mean free path of photon transport beneath the mouse surface on the *z* = 7.3 mm plane where the heart centered.  Figure 6 illustrates the reconstruction results (which are normalized to the intensity of the actual target) obtained by the SCP-Kaczmarz and the Kaczmarz method after 100 iterations, where the actual heart inside the body is hidden for clarity. We also plotted the recovered intensity at each node of the discretized atlas torso, as shown in Figure 7, where the *x*-axis denotes the node index and the *y*-axis denotes the intensity. The relative deviation, Dice coefficient, and sparsity are illustrated in Figure 8. Although the two methods get different results, the sparsity values are the same after 40 iterations. The computational time of the SCP-Kaczmarz and Kaczmarz is 80 seconds and 81 seconds, respectively, and both the memory usages are about 565 MB.

#### 2.4.2. Reconstruction of Multiple Fluorescent Targets

**Figure 9:**
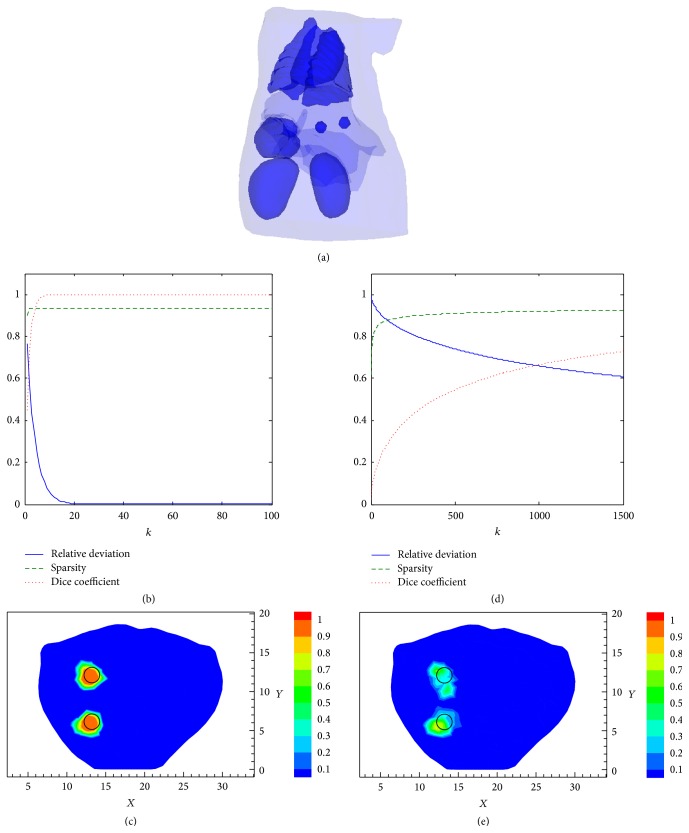
Reconstruction results of two targets. (a) The actual targets. (b) The relative deviation, sparsity, and Dice coefficient obtained by the SCP-Kaczmarz method. (c) The 2D slices at *z* = 16.5 mm obtained by the SCP-Kaczmarz algorithm. (d) The relative deviation, sparsity, and Dice coefficient obtained by the Kaczmarz method. (e) The 2D slices at *z* = 16.5 mm obtained by the Kaczmarz algorithm.

In this section, we used the proposed SCP-Kaczmarz method to recover multiple targets. As shown in Figure 9(a), two spherical fluorescent targets with radius of 1 mm were placed at “13 mm, 12 mm, and 16.5 mm” and “13 mm, 6 mm, and 16.5 mm” in the liver of the digital mouse. Figure 9(b) illustrates the relative deviation (blue solid line), sparsity (green dashed line), and Dice coefficient (red dotted line) obtained by the SCP-Kaczmarz method and Figure 9(c) is the recovered slices at *z* = 16.5 mm after 100 iterations where the black circles denote the actual targets. The corresponding results obtained by the Kaczmarz method are plotted in Figures 9(d) and 9(e). It can be seen that about 1500 iterations are needed for the Kaczmarz algorithm to get satisfactory results. The time for the SCP-Kaczmarz to run 100 iterations is 72 seconds and that for the Kaczmarz to run 1500 iterations is 1209 seconds and the corresponding memory cost is about 559 and 557 MB, respectively.

**Figure 10:**
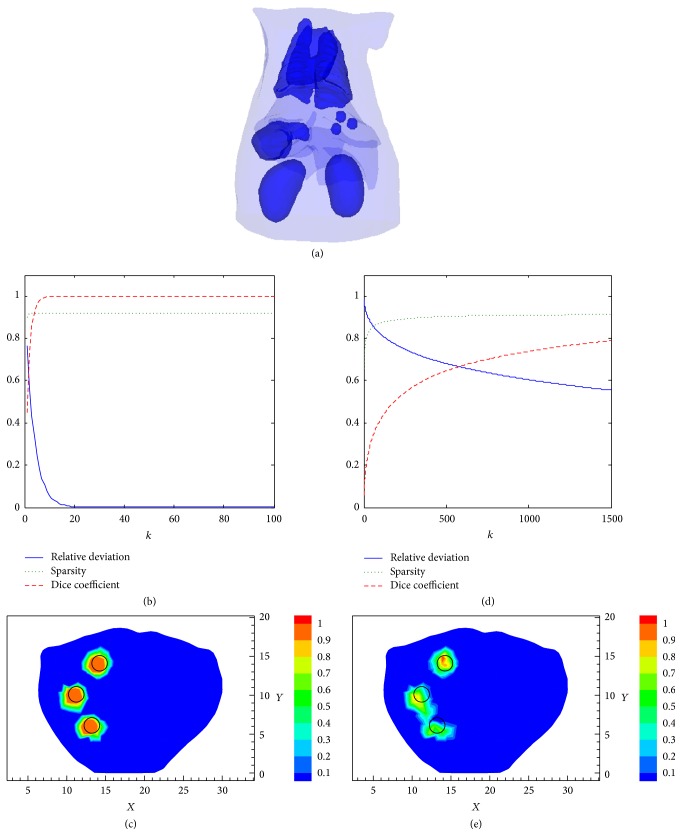
Reconstruction results of three targets. (a) The actual targets. (b) The relative deviation, sparsity, and Dice coefficient obtained by the SCP-Kaczmarz method. (c) The 2D slices at *z* = 16.5 mm obtained by the SCP-Kaczmarz algorithm. (d) The relative deviation, sparsity, and Dice coefficient obtained by the Kaczmarz method. (e) The 2D slices at *z* = 16.5 mm obtained by the Kaczmarz algorithm.

To further test the ability of the proposed method to distinguish multiple targets, we considered three fluorescent spheres with radius of 1 mm placed in the liver of the digital mouse. As shown in Figure 10(a), the three fluorescent targets were centered at “11 mm, 10 mm, and 16.5 mm”; “14 mm, 14 mm, and 16.5 mm”; and “13 mm, 6 mm, 16.5 mm,” respectively. The SCP-Kaczmarz results after 100 iterations are demonstrated in Figures 10(b) and 10(c), and the Kaczmarz results after 1500 iterations are demonstrated in Figures 10(d) and 10(e). The actual targets are indicated by black circles in Figures 10(c) and 10(e). The time cost for 100 SCP-Kaczmarz iterations and 1500 Kaczmarz iterations is about 78 seconds and 1327 seconds, respectively. The memory consumption for the two methods is about 555 MB. The results show that the three targets can be distinguished by the two methods. However, the proposed new method performs better both in accuracy and in convergence rate compared with the Kaczmarz method.

### 2.5. Phantom and In Vivo Experiments

In this section, our homemade dual-modality FMT-Micro-CT imaging system [19] was used to perform the phantom and in vivo experiments. Two phantom experiments were conducted. In the first experiment, a 20 mm cubic phantom made from polyoxymethylene was placed on the rotational stage of the imaging system. A small hole with 1 mm radius and 2 mm height was drilled at “15 mm, 7 mm, and 9.5 mm” in the phantom. 40 *μ*M of Cy5.5 solution was emplaced in the hole to be used as the fluorescent target. A 671 nm CW laser was used as the point source to excite the Cy5.5 solution on each side of the cubic phantom and a 40 nm bandpass filter centered at 720 nm was set before an EMCCD camera to collect the fluorescence signal on the phantom surface. The optical parameters for excitation and emission wavelengths are *μ*_*ax*_ = 0.0134 mm^−1^ and *μ*_*sx*_ = 9.3 mm^−1^ and *μ*_*am*_ = 0.0114 mm^−1^ and *μ*_*sm*_ = 10.1 mm^−1^, respectively. In the second experiment, a cylinder phantom with 10 mm radius and 30 mm height was used. Two holes with 1 mm radius and 5 mm height were drilled in the cylinder phantom. The two holes were centered at “5 mm, 4 mm, and 15 mm” and “5 mm, −4 mm, and 15 mm,” respectively. Both holes were filled with 40 *μ*M of Cy5.5 solution. The 671 nm CW laser was used as the point source. Five excitation points were set equivalently along the right half side of the phantom on *z* = 15 mm plane.

**Figure 11:**
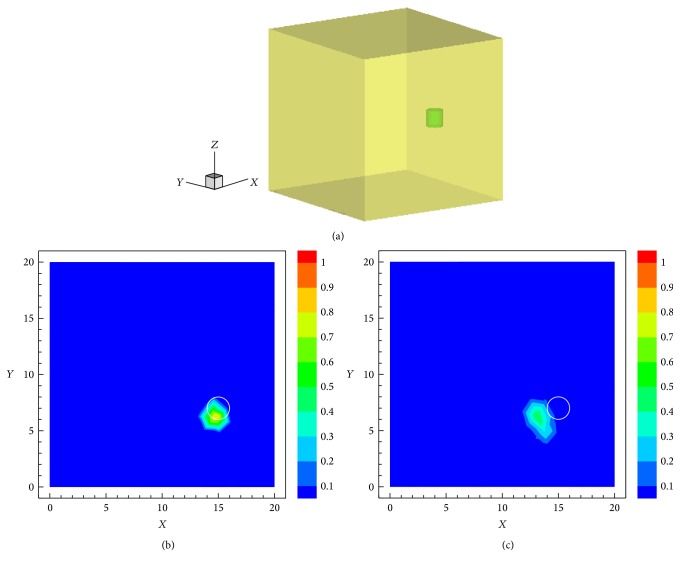
Reconstruction of single target in the cubic phantom. (a) The actual fluorescent target. (b) The normalized result by the SCP-Kaczmarz technique on plane *z* = 9.5 mm. (c) The normalized result by the Kaczmarz technique on plane *z* = 9.5 mm.

The cubic phantom is illustrated in Figure 11(a), where the green cylinder denotes the Cy5.5 solution. The initial sparsity value was set to be 0.8 for the first iteration. Figures 11(b) and 11(c) show the normalized results at *z* = 9.5 mm by the SC-Kaczmarz and the Kaczmarz method after 200 iterations, respectively, where the true target is indicated by the white circle and the location errors are about 0.7 mm and 1.9 mm, respectively. Compared with the Kaczmarz result, the intensity of the SCP-Kaczmarz result is larger due to the fact that the latter one distributes more sparsely. The time cost is 55 seconds and 58 seconds and the memory cost is about 100 MB.

**Figure 12:**
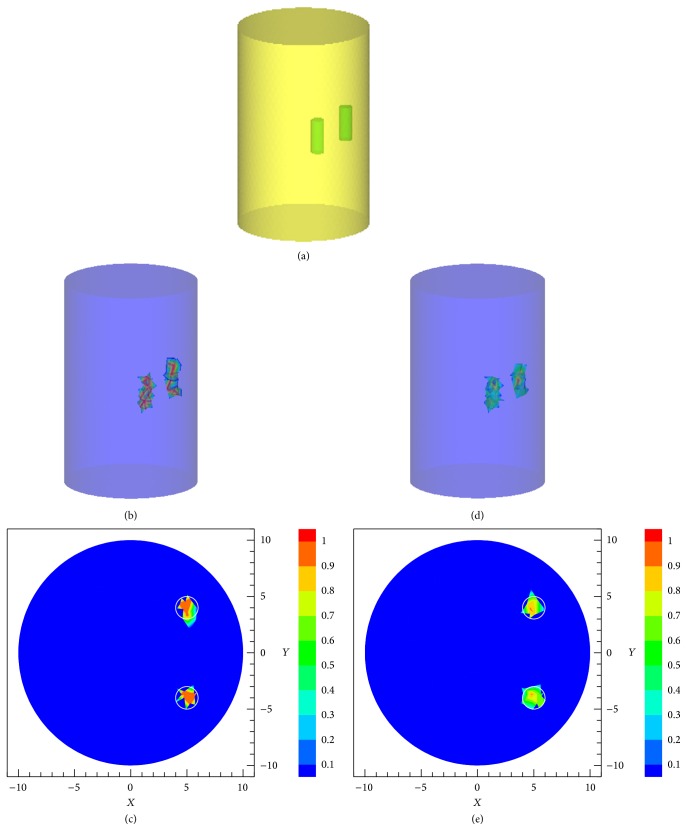
Reconstruction of two targets in the cylinder phantom. (a) The actual targets. (b) The 3D targets and (c) the 2D slices at *z* = 15 mm recovered by the SCP-Kaczmarz method after 100 iterations. (d) The 3D targets and (e) the 2D slices at *z* = 15 mm recovered by the Kaczmarz method after 500 iterations.

The cylinder phantom is shown in Figure 12(a), where the green cylinders denote the Cy5.5 solution. Figures 12(b) and 12(c) show the reconstructed 3D targets and 2D slices at *z* = 15 mm by the SCP-Kaczmarz method after 100 iterations. To get acceptable results, 500 iterations were needed for the Kaczmarz method. Figures 12(d) and 12(e) show the reconstructed 3D targets and 2D slice at *z* = 15 mm by the Kaczmarz method after 500 iterations. 167 seconds is cost for the SCP-Kaczmarz algorithm to iterate 100 times and 862 seconds is needed for the Kaczmarz algorithm to iterate 500 times. The memory used by the two methods is about 600 MB, where the phantom is discretized into 7851 nodes for inverse reconstruction.

**Figure 13:**
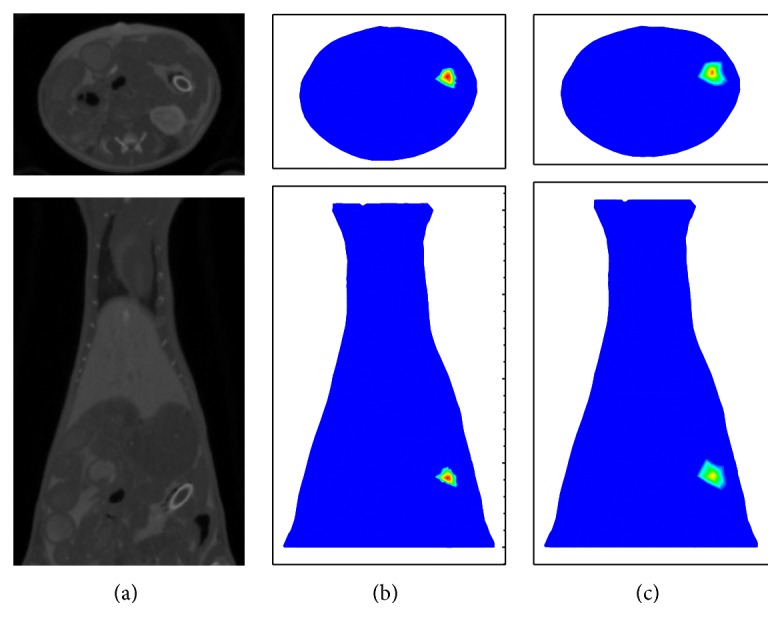
In vivo experiment results. (a) CT slices where the glass tube is the true target. (b) Recovered slices by the SCP-Kaczmarz method. (c) Recovered slices by the Kaczmarz method.

The proposed SCP-Kaczmarz method was also used to recover the fluorescent target from the in vivo small animal experimental data [32]. In the experiment, the fluorescent target was made of a glass tube full of 4000 nM Cy5.5 solution and was implanted into the abdomen of an adult BALB/C mouse. A 671 nm CW point laser was used to excite the target at four positions sequentially and the optical signal on the mouse surface was collected. After the optical images acquisition, the anesthetized mouse was scanned by the Micro-CT subsystem. The reconstructed CT slices were segmented into five components (heart, lungs, liver, kidneys, and muscle) and used to provide prior structural information for the FMT inverse problem. The sparsity value was set to be 0.7 for the first iteration. The in vivo experiment results are shown in Figure 13, where (a) is the CT slices, (b) is the recovered slices by the SCP-Kaczmarz, and (c) is the recovered slices by the Kaczmarz method. The top row in Figure 13 illustrates the cross views and the bottom row demonstrates the coronal views. With Micro-CT, the center of the glass tube is around “21.1, 27.8, and 7.4” mm. The center of the recovered target by SCP-Kaczmarz is about 1.4 mm away from the glass tube center after 100 iterations. The center of the recovered target by Kaczmarz is about 1.7 mm away from the glass tube center after 150 iterations. As sparsity constraint is applied, we can see from Figure 10 that the reconstructed target by SCP-Kaczmarz is sparser and the amplitude is larger than the target recovered by Kaczmarz. The time cost is 50 seconds and 80 seconds for the SCP-Kaczmarz method (100 iterations) and the Kaczmarz method (150 iterations), respectively, and the memory used in both is about 270 MB.

## 3. Discussion and Conclusion

In this work, we propose a sparsity-constrained preconditioned Kaczmarz method to solve the inverse problem in FMT. First, a preconditioner is computed to minimize the correlation between the rows of the FMT forward matrix, then the classical Kaczmarz method is used to solve the preconditioned inverse problem, and finally the large elements of the Kaczmarz solution are kept and the other elements are set to zero to make the result satisfy a preset sparsity value. The threshold value is obtained by solving a minimization problem using linear searching strategy. The performance of the proposed algorithm is demonstrated by numerical simulation and phantom and in vivo experiments. In numerical simulation, both small target and big target can be recovered with high accuracy by the proposed method. As the correlation between the rows of the forward matrix has been minimized, the SCP-Kaczmarz method converges to the true solution rather faster than the classical Kaczmarz method. In the phantom and in vivo experiment, the proposed algorithm shows performance improvement both in location accuracy and in convergence speed relative to the classical Kaczmarz technique. Furthermore, experiments with two targets and three targets were performed. The results show that both the methods can get satisfactory results. However, we find that the Kaczmarz method converges rather slowly when recovering more than one target, while the convergence of the proposed SCP-Kaczmarz is not affected by the number of targets. The reason may be that the correlation between the rows of the forward matrix when multiple targets are present is stronger compared to the case when one target is present. And the stronger correlation leads to slower convergence of the Kaczmarz method. After preconditioning, the correlation is minimized and hence the convergence remains the same for different number of targets. The computational time and the memory usage are also calculated. The results show that, under the same number of iterations, the Kaczmarz method runs a little faster than the SCP-Kaczmarz method, and they consume similar memory. That is because the memory is mainly used to store the preconditioned or the original forward matrix which are of the same size.

A parameter, named the wanted sparsity value, should be predetermined when using the SCP-Kaczmarz method. As the number of nodes in the imaging domain is known, we can estimate the number of nodes the fluorescent target covers and hence get a sparsity value by (9). However, we found that the sparsity value has little effect on the reconstruction result in our experiment. It is known to us that the singular value decomposition of the forward matrix, needed for the proposed method to calculate the preconditioner, is computationally expensive. Fortunately, the SVD only needs to be performed for one time and can be done before the iteration starts. No additional computation load will be caused when the proposed method iteratively solves the preconditioned FMT inverse problem. In numerical simulation, as the surface data was obtained based on the diffusion approximation (DA) model, which is also used in solving the inverse problem, the proposed algorithm performs well to reconstruct the intensity and the shape of the fluorescent target. For real experiment, we do not know actually the light transport model. So there is a mismatch between the true model and the diffusion approximation, and the proposed method does not perform as well as it does in the simulation experiment. In the future, we will focus on light transport model based on higher order approximation (e.g., the SP_N_ approximation) and perform in vivo experiment with multiple targets to further investigate the proposed method.

## Figures and Tables

**Table 1:** Optical parameters of the mouse organs [19].

Tissue	*μ*_*ax*_ (mm^−1^)	*μ*_*sx*_′ (mm^−1^)	*μ*_*am*_ (mm^−1^)	*μ*_*sm*_′ (mm^−1^)
Muscle	0.0052	1.08	0.0068	1.03
Heart	0.0083	1.01	0.0104	0.99
Lungs	0.0133	1.97	0.0203	1.95
Liver	0.0329	0.70	0.0176	0.65
Kidneys	0.0660	2.25	0.0380	2.02
Stomach	0.0114	1.74	0.0070	1.36
